# Predictive Risk Mapping of Schistosomiasis in Madagascar Using Ecological Niche Modeling and Precision Mapping

**DOI:** 10.3390/tropicalmed7020015

**Published:** 2022-01-19

**Authors:** Mark A. Deka

**Affiliations:** Centers for Disease Control and Prevention (CDC), 4770 Buford Hwy NE, Atlanta, GA 30341, USA; pmu5@cdc.gov

**Keywords:** disease mapping, geographic information science, schistosomiasis, precision public health, ecological niche modeling

## Abstract

Schistosomiasis is a neglected tropical disease (NTD) found throughout tropical and subtropical Africa. In Madagascar, the condition is widespread and endemic in 74% of all administrative districts in the country. Despite the significant burden of the disease, high-resolution risk maps have yet to be produced to guide national control programs. This study used an ecological niche modeling (ENM) and precision mapping approach to estimate environmental suitability and disease transmission risk. The results show that suitability for schistosomiasis is widespread and covers 264,781 km^2^ (102,232 sq miles). Covariates of significance to the model were the accessibility to cities, distance to water, enhanced vegetation index (EVI), annual mean temperature, land surface temperature (LST), clay content, and annual precipitation. Disease transmission risk is greatest in the central highlands, tropical east coast, arid-southwest, and northwest. An estimated 14.9 million people could be at risk of schistosomiasis; 11.4 million reside in rural areas, while 3.5 million are in urban areas. This study provides valuable insight into the geography of schistosomiasis in Madagascar and its potential risk to human populations. Because of the focal nature of the disease, these maps can inform national surveillance programs while improving understanding of areas in need of medical interventions.

## 1. Introduction

Schistosomiasis is an acute and chronic parasitic infection caused by trematodes of the genus *Schistosoma* [1]. The disease is widespread throughout sub-Saharan Africa (SSA), where an estimated 800 million people are at risk of infection [1,2]. Globally, schistosomiasis is endemic in 78 countries [1]. Annually, schistosomiasis is estimated to account for between 200,000 and 535,000 deaths in SSA alone [1,3]. The intermediate hosts of human Schistosoma during the asexual stage belong to three freshwater snail genera, *Biomphalaria*, *Bulinus,* and *Oncomelania* [4,5,6]. In impoverished, rural areas, the disease is prevalent in agricultural and fishing communities and among those who take part in everyday domestic, occupational, and recreational activities within waterbodies. Children are especially at risk of the disease when swimming or playing in infected water [7,8]. After Malaria, schistosomiasis is ranked as the second most devastating parasitic disease in terms of its socioeconomic impact on people [7]. Like many neglected tropical diseases (NTDs), schistosomiasis is associated with regions of high poverty and poor sanitation [9,10].

In Madagascar, the burden of schistosomiasis is high [3], with 107/144 districts reporting the disease as endemic in 2016 [11,12]. Only 11% of the population has access to improved methods of sanitation, and 44% practice open defecation [13,14]. It is estimated that 52.1% of the total population is infected with schistosomiasis, representing the fifth highest globally [15]. Both *Schistosoma haematobium* and *Schistosoma mansoni* are found in Madagascar. *Schistosoma haematobium,* which causes urogenital schistosomiasis, is predominately found in the northern and western districts. Likewise, *Schistosoma mansoni,* which causes an intestinal version of the disease, is prevalent in the eastern and southern districts. Co-endemicity between these species is noted in the north-central and southwest [11,12]. The burden of the disease is felt considerably by school-aged (SAC) and pre-school-aged children. National control campaigns offering mass drug administration (MDA) of praziquantel for children aged 5–15 often struggle with logistical challenges due to the remoteness of some endemic regions [12]. Much of the Malagasy population in rural areas have limited access to government-run primary health care centers, which often lack physicians and laboratory testing equipment [16].

With the incorporation of geospatial technologies into fields like public health, epidemiology, and disease ecology, our knowledge of the spatial patterns of disease has increased significantly in recent decades. The use of geographic information systems (GIS) and the adoption of remotely sensed (RS) data products have been widely used in disease mapping and epidemiology [17]. As an essential tool of 21st-century medical geographers, GIS provides estimates of the spatial risk of disease at multiple scales of analysis, facilitating public health interventions [18]. Disease mapping and spatial modeling are increasingly utilized to guide intervention strategies, derive health metrics, and enhance epidemiological understanding of humans and their environment [19]. GIS-based disease mapping is primarily focused on identifying the locations of disease occurrence, patterns of diffusion, and environmental risk factors [20,21]. Literature on the use of these techniques for studies on schistosomiasis ranges geographically from China [22], Brazil [23], Nigeria [24], The Philippines [25], sub-Saharan Africa [26], and Ethiopia [27].

To date, no studies have attempted to examine the geography of schistosomiasis in Madagascar and its potential risk to human populations. To fill this gap, using an ecological niche modeling (ENM) and precision mapping approach [28], this study sought to (i) develop a model of environmental suitability for the disease (ii) and to map the potential disease exposure risk. High-resolution maps are necessary due to the focal nature of schistosomiasis. These risk maps will provide valuable eco-epidemiological information to inform decision-makers in effectively allocating resources for targeted prevention and control measures.

## 2. Materials and Methods

### 2.1. Study Area

**Figure 1:**
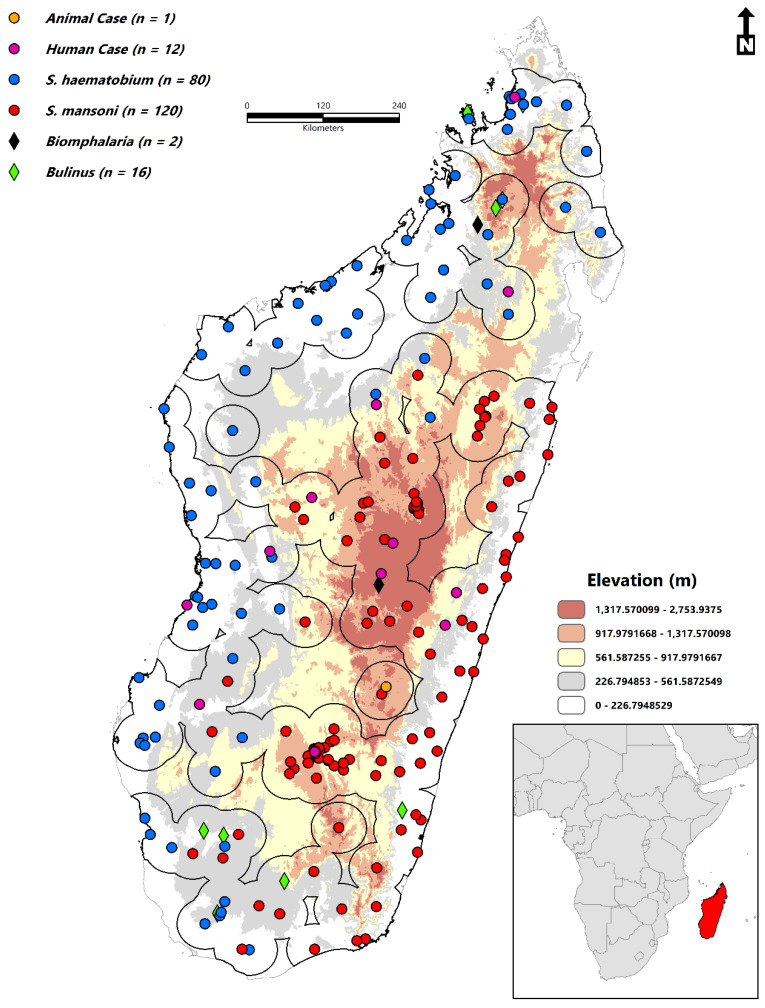
Geographic distribution of occurrence data (*n* = 231) in comparison to the predominant topographic characteristics of Madagascar. The model calibration area (**M**) is visualized as 40-km buffers (black).

Madagascar (Figure 1) (18.7669° S, 46.8691° E) lies approximately 400 km (250 miles) off the coast of East Africa and is the world’s second-largest island nation (587,041 km square) after Indonesia. The estimated population in 2021 is 28,427,328 [29]. According to the International Monetary Fund (IMF), the per capita GDP (nominal) is estimated at $471 per person (2019) [30]. Madagascar ranked 164th in the world in 2019 according to the United Nations Development Programme (UNDP) Human Development Index (HDI) [31]. Eight neglected tropical diseases (NTDs) are considered endemic on the island: schistosomiasis (SCH), soil-transmitted helminths (STH), lymphatic filariasis (LF), dengue fever, rabies, leprosy, tungiasis, and plague [32].

### 2.2. Occurrence Data

Geographic records of both *Schistosoma haematobium* (*n* = 80) and *Schistosoma mansoni* (*n* = 120) were collected from the Global Atlas of Helminth Infections (GAHI) (http://www.thiswormyworld.org/) (access date: 2 November 2021) [33] and the World Health Organization (WHO) [34]. Supplementing these data were literature on confirmed human (*n* = 12) and animal (*n* = 1) cases [35,36,37,38,39,40,41,42,43,44] extracted from a literature search in Google (www.google.com) (access date: 2 November 2021), Google Scholar (https://scholar.google.com/) (access date: 2 November 2021), and PubMed (https://pubmed.ncbi.nlm.nih.gov/) (access date: 2 November 2021). No time range was specified or article type limits. Search terms included “schistosomiasis Madagascar”, schistosomiasis Madagascar animals”, “schistosomiasis Madagascar humans”. Bulinus (*n* = 16) and Biomphalaria (*n* = 2) occurrence records were also included to account for the immediate host stage. These data were collected from the Global Biodiversity Information Facility (GBIF) [45,46]. In total, 231 records were compiled from 1921–2021 (animal case (*n* = 1), human case (*n* = 12), *S. haematobium* (*n* = 80), *S. mansoni* (*n* = 120), *Bulinus* (*n* = 16), *Biomphalaria* (*n* = 2)). Please see the Supplementary Materials for the complete list of occurrence data and their geographic information (Table S1). Before the ecological niche modeling stage, the occurrence data were cleaned by removing duplicate records. Sampling bias needed to be accounted for [47,48] because records were not sampled evenly across the study area. The ‘spThin’ [49] R programming language package (version 4.1.2–R Core Team) [50] removed duplicate points at a distance threshold of 25 km. The final dataset thus featured 127 spatially independent records. The model calibration areas were established based on the recommendation of Barve and colleagues [51]. The accessible area or the **M** region [52] within the BAM (Biotic–Abiotic-Movement) Framework was defined by 40-km buffers surrounding the filtered occurrence data.

### 2.3. Environmental Variables

Like other neglected tropical diseases (NTDs), schistosomiasis is influenced by various environmental factors that govern the persistence of the disease and the survival of snail vectors [53,54,55,56]. To characterize the present climatic conditions, bioclimatic variables were obtained from the WorldClim dataset (1970–2000) (v2.1) (https://worldclim.org/) (access date: 2 November 2021) [57] at the 1-km resolution (30-arc seconds). Before modeling, several variables were excluded from the analysis (bio8, bio9, bio18, bio19) due to known spatial artifacts affecting the ecological niche modeling process [58]. Gridded soil data representing the predominant silt, sand, and clay content were obtained from the International Soil Reference and Information Centre (ISRIC) (https://soilgrids.org/) (access date: 2 November 2021) at a depth of 0–5 cm (1-km). Topographic data were extracted from a digital elevation model (DEM) representing the mean elevation of Madagascar. This data was downloaded from EarthEnv (https://www.earthenv.org/) (access date: 2 November 2021) [59] and represented an enhanced model called the Global Multi-resolution Terrain Elevation Data (GMTED2010).

To explore the potential effects of vegetation, surface energy, and water balance, moderate resolution imaging spectroradiometer (MODIS) (National Aeronautics and Space Administration (NASA)) monthly mean enhanced vegetation index (EVI) and mean eight-day land surface temperature (LST) datasets were obtained from the WorldGrids data archive [60] (1-km). The EVI is an optimized vegetation index that enhances signal sensitivity in high biomass regions and improves vegetation monitoring capabilities. Land surface temperature (LST) is simply the radiative skin temperature of land derived directly from infrared radiation. It is a useful variable because it contains a mixture of bare soil and temperature data. Also included were two sociodemographic variables which could potentially contribute to the disease transmission risk in rural areas: the accessibility to cities (1-km) (2015) [61], and nighttime lights satellite imagery (2013) (1-km) (National Oceanic and Atmosphere Administration (NOAA)) (https://ngdc.noaa.gov/eog/dmsp/downloadV4composites.html) (access date: 2 November 2021) [62]. The distance to water bodies was also included in the analysis. This variable served as a spatial risk factor for humans and the habitat for the intermediate freshwater snail hosts. The dataset was created by applying the Euclidean distance analysis tool in ArcGIS 10.8.1 (Environmental Systems Research Institute, RedLands, CA, USA) at a maximum distance threshold of approximately 16 km (25,749.5 m) with an output cell size of 1-km. These water features were obtained from the website DIVA-GIS (https://www.diva-gis.org/) (access date: 2 November 2021).

### 2.4. Variable Selection

A pairwise Pearson’s correlation coefficient (PCC) [63] analysis was done using the R programming package (version 4.1.2–R Core Team), ‘ntbox’ v0.5.1.4 [64]. This step in the pre-modeling process reduced multicollinearity between the predictor variables and only variables with a value less than ±0.75 were retained. Pearson’s correlation coefficient is defined as the covariance of two variables divided by the product of their standard deviations [63]. The final set of candidate variables were: annual mean temperature (bio1), Isothermality (bio3), temperature seasonality (bio4), annual precipitation (bio12), precipitation seasonality (bio15), accessibility to cities, clay and silt content, distance to water, land surface temperature (LST), enhanced vegetation index (EVI), and NOAA nighttime lights (Table 1).

### 2.5. Ecological Niche Modeling

An ensemble ecological niche model (ENM) was developed with the R programming language (version 4.1.2–R Core Team) [50] package ‘biomod2’ [65]. The ecological niche methodology consists of developing a predictive model of the geographic distribution of species based on their known environmental requirements and occurrence data [66]. Ecological niche modeling (ENM) has increasingly been applied in a public health context to characterize the ecological conditions that support disease agents and promote their transmission [27,67,68]. In total, four algorithms were chosen for the ENM process: Generalized Boosted Models (GBM) [69], Generalized Linear Models (GLM) [70], Random Forest (RF) [71], and Multiple Adaptive Regression Splines (MARS) [72]. Pseudoabsence data (1:2 ratio = 254 PA) were generated with the ‘surface-range envelope’ model (similar to BIOCLIM). Here, random points were selected from all points outside the suitable area estimated by a rectilinear surface envelope from the presence sample (quantile = 0.025–95% CI) [65]. Each algorithm was run 25 times (4 algorithms × 25 replicates = 100 models), with 80% of the data allocated for training and 20% used for testing. Please see Supplementary Materials (Image S1) for the corresponding environmental variable response plots.

For each algorithm, the area under the curve (AUC) of the receiver operating characteristic (ROC) [66] and the true skill statistic (TSS) [73] were applied to evaluate the predictive performance of each metric. The AUC differentiates between negative and positive values and ranges from 0 to 1, with high values (greater than 0.70) indicating better predictive potential. On the contrary, the TSS is a prevalence-independent measure calculated as sensitivity + specificity − 1, with values ranging from −1 (random) to 1 (perfect model performance. The variable importance of the non-correlated variables was based on a decrease in accuracy and on correlating the fitted data with the randomly permitted values [74]. Models with mean AUC values greater than 0.70 were combined based on the estimated weighted sum of predictions (weighted mean). The coefficient of variation (CV) between values served as a measure of overall model uncertainty. The final ensemble was also converted to a binary outcome (i.e., suitable, or non-suitable) based on a cut-off value which best represented the trade-off between sensitivity, specificity, and accuracy [75].

### 2.6. Estimating Zones of Exposure Risk and the At-Risk Population

To map the disease transmission risk associated with schistosomiasis, two components were combined: (1) the potential abundance of the disease, the ensemble ecological niche model (threat), and (2) gridded human population density data (vulnerability) [76,77]. The human population density grid (2020) with a spatial resolution of 1-km was obtained from the WorldPop mapping project (www.worldpop.org) (access date: 2 November 2021). To estimate exposure risk zones, a three-step process was applied. First, the population density data was classified into four categories: null (0–1 persons/km^2^), low (>1–10 persons/km^2^), medium (>10–100 persons/km^2^), and high (>100 persons/km^2^). Numerical values were then assigned to each of these categories: null = 0, low = 1, medium = 2, high = 3. Second, the weighted mean model was reclassified into four categories: null, low, medium, and high with an equal interval classification type. Third, both reclassified grids were combined in the Raster Calculator tool in ArcGIS 10.8.1 (Environmental Systems Research Institute, RedLands, CA, USA).

The final output map featured exposure risk zones ranging from very low, low, medium, high, and very high [76]. Estimates on the total number of people living in suitable areas were then obtained by overlaying the binary output map (i.e., suitable, or non-suitable) with human population data representing the total count of persons per pixel value (1-km) (2020) (www.worldpop.org) (access date: 2 November 2021). The estimated at-risk population was then split into two classification schemes: urban-rural based on boundaries established from The Global Rural-Urban Mapping Project (GRUMP) v1 (CIESIN) (Global Rural-Urban Mapping Project (GRUMP), v1|SEDAC (columbia.edu) (access date: 2 November 2021)).

## 3. Results

**Figure 2:**
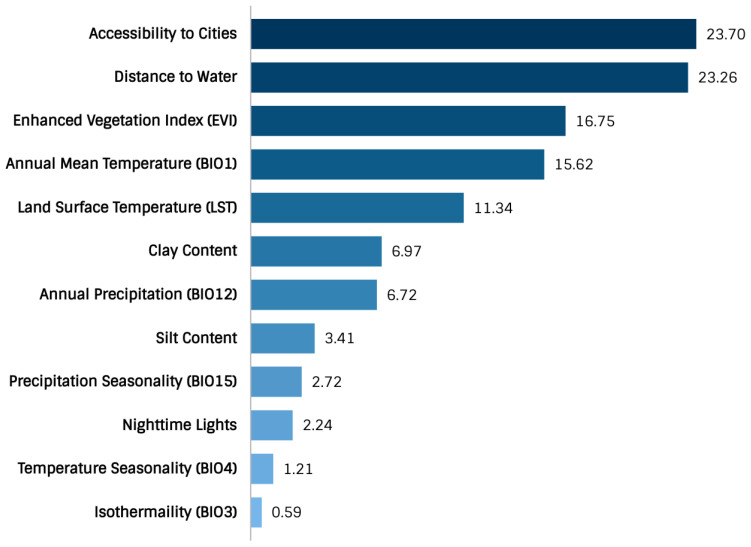
Variable contribution for the weighted mean suitability model.

**Figure 3:**
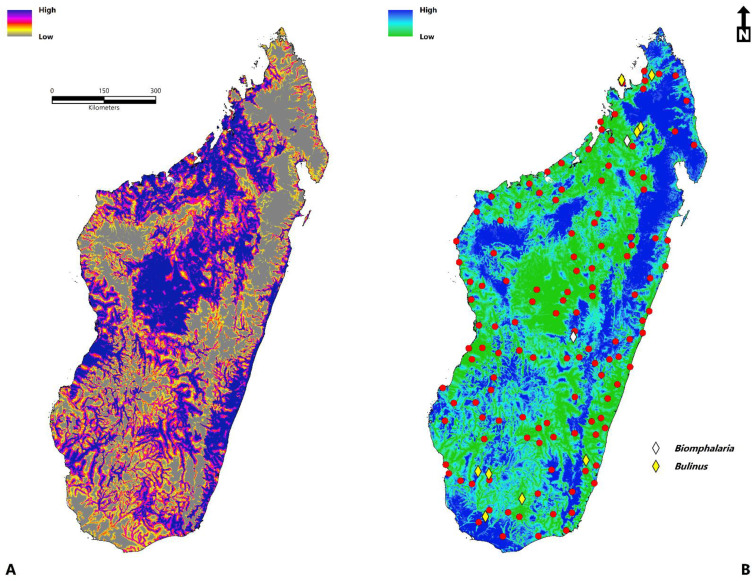
Environmental suitability of schistosomiasis in Madagascar (**A**). The estimated weighted sum of predictions (weighted mean) (**B**). Model uncertainty based on the coefficient of variation (CV). The filtered occurrence records (red) are superimposed, including the aquatic snails *Biomphalaria* and *Bulinus*.

In this study, two hundred thirty-one records (Figure 1) were collected, all of which spanned a temporal period of 100 years from 1921–2021. When documented at the regional administrative level (Level 2-Database of Global Administrative Areas (https://gadm.org/)) (access date: 2 November 2021), 17% (*n* = 39) of all occurrences were documented in Ihorombe, 10% (*n* = 24) in Atsimo-Andrefana, 9.5% (*n* = 22) in Menabe, 7% (*n* = 16) in Diana, 7% (*n* = 16) in Sofia, and 6.5% (*n* = 15) in Analamanga. The total area predicted to be suitable for schistosomiasis in Madagascar is 264,781 km^2^ (102,232 sq miles). Variables with the highest contribution to the ecological niche model (Figure 2) were the accessibility to cities (23.70), distance to water (23.26), enhanced vegetation index (EVI) (16.75), annual mean temperature (bio1) (15.62), land surface temperature (LST) (11.34), clay content (6.97), annual precipitation (bio12) (6.72), silt content (3.41), precipitation seasonality (bio15) (2.72), nighttime lights (2.24), temperature seasonality (bio4) (1.21), and Isothermality (bio3) (0.59). The predicted environmental suitability of schistosomiasis and the associated model uncertainty are presented in Figure 3.

**Figure 4:**
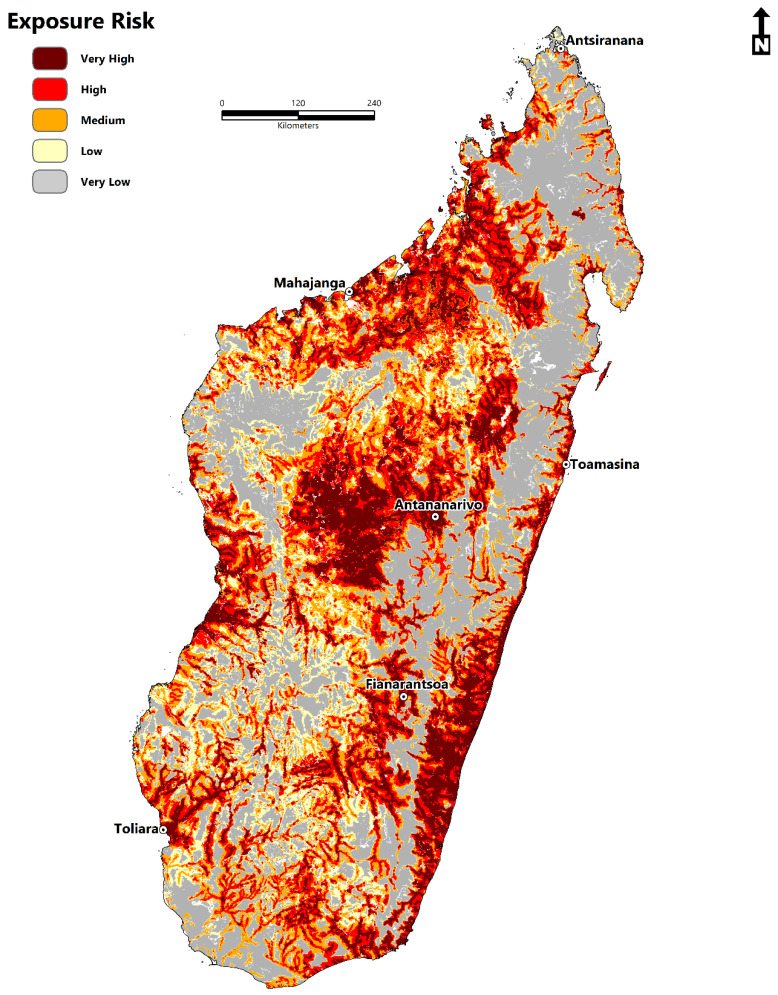
Schistosomiasis disease exposure risk. The color scale from orange to dark red corresponds to medium, high, and very high exposure risk, while values from yellow to grey represent low–very low risk.

The spatial distribution is widespread throughout the island, particularly in the sub-arid southwest regions of Atsimo-Andrefana, Androy, Atsimo-Atsinana, Ihorombe, Menabe, and the east coast within Vatovavy Fitovinany, Atsinanana, and Analanjirofo. Suitability within the sub-humid central plateau is present in and around Antananarivo, Antsirabe, and Fianarantsoa. Similarly, the north-western region has suitable areas in Boeny and Sofia, the far northeast and west, and to a limited extent in Diana and Sava. The risk associated with schistosomiasis to human populations (Figure 4) is at its greatest geographic extent within the sub-humid central highland region, humid tropical eastern coast, dry-arid southwest, northwest, and, to a lesser extent, the far north and east. As expected, much of the high and very high-risk areas for disease transmission are concentrated in and around the urban areas of Antananarivo, Fianarantsoa, and the coastal cities of Toamasina, Toliara, Mahajanga, and Antsiranana. The risk in rural areas, although less pronounced because of a lower population density, is still significant, particularly in areas throughout the southwest, western coast, and northwest.

**Figure 5:**
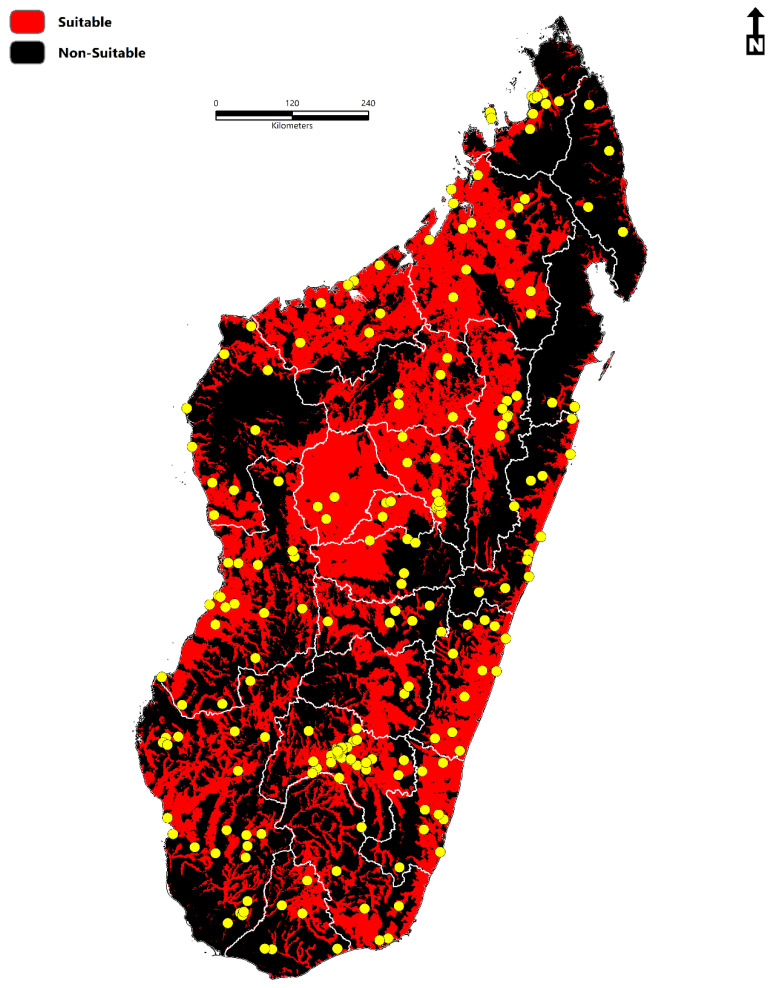
The environmental suitability of schistosomiasis in Madagascar based on a binary threshold value of 0.478. Level 2 classifications represent administrative boundaries according to the Database of Global Administrative Areas (https://gadm.org/) (access date: 2 November 2021). Yellow dots represent the study occurrence data (*n* = 231).

The average ROC scores for the chosen algorithms (GBM, GLM, RF, MARS) were high, with all four averaging ROC scores ≥ 0.80. The most robust predictive performance was displayed between the Generalized Boosted Model (GBM) (0.86) and Random Forest (RF) (0.84) algorithms, while Multiple Adaptive Regression Splines (MARS) and Generalized Linear Models (GLM) had average ROC scores of 0.81 and 0.80 respectively. When compared, the ROC values for all models ranged from a minimum of 0.71 to a high of 0.98, while the TSS values ranged from a minimum of 0.34 to a high of 0.97. The estimated human population at risk of schistosomiasis (2020) is 14,972,194 (Figure 5). Of these 14.9 million, 3,545,616 live in urban areas, and 11,426,578 live in rural areas. The estimated population at risk represents roughly 53% of Madagascar’s total population.

## 4. Discussion

Historically, disease mapping has been considered an essential tool when examining the connection between place, space, and human health. These methods have evolved markedly in recent decades and have become one of the most critical GIS technologies in developing improved disease surveillance systems [78,79]. GIS-based disease mapping has been applied successfully in previous studies examining the geography of neglected tropical diseases (NTDs) [80,81,82,83]. In this study, an ecological niche modeling and precision mapping approach were combined to estimate the environmental suitability of schistosomiasis and the risk of disease transmission to humans. These models were developed by relating the location of occurrence data with sociodemographic and environmental variables. The ecological niche model represented the relative environmental risk of schistosomiasis and the corresponding level of model uncertainty across Madagascar.

The present study shows that the suitability of schistosomiasis in Madagascar and the risk to human populations has a broad geographic distribution across the island and is at its most significant in the southwest regions of Atsimo-Andrefana, Androy, Atsimo-Atsinana, Ihorombe, Menabe, and the eastern areas of Vatovavy Fitovinany, Atsinanana, and Analanjirofo. Within the sub-humid central plateau region, suitability is prevalent in Antananarivo, Antsirabe, and Fianarantsoa. While, in the northwest, suitable areas are distributed in Boeny and Sofia, and to a limited extent, coastal regions in Diana and Sava.

When mapped at the regional administrative level, 17% of all occurrences were in Ihorombe (*n* = 39), 10% (*n* = 24) in Atsimo-Andrefana, 9.5% (*n* = 22) in Menabe, and 7% were found in Diana (*n* = 16), respectively. Variables of significance to the ecological niche model were the accessibility to cities (23.70), distance to water (23.26), enhanced vegetation index (EVI) (16.75), annual mean temperature (bio1) (15.62), land surface temperature (LST) (11.34), clay content (6.97), annual precipitation (bio12) (6.72), silt content (3.41), precipitation seasonality (bio15) (2.72), NOAA nighttime lights (2.24), temperature seasonality (bio4) (1.21), and Isothermality (bio3) (0.59). In total, 14,972,194 people are at risk of schistosomiasis, with an estimated 3,545,616 living in urban areas and an additional 11,426,578 in rural areas. The total population at risk constitutes roughly 53% of the country’s total population. Estimates of the human population at risk were obtained by converting the weighted sum of predictions to a binary (i.e., suitable, or non-suitable) model, which best represented a cut-off value that balanced model sensitivity, specificity, and accuracy.

This study additionally applied a precision mapping approach to quantify and map the exposure risk to schistosomiasis. Precision mapping has its roots in the perspective of precision public health, integrating geolocated information and maps to pinpoint regions of elevated health risk with high degrees of accuracy [77,84,85,86]. Public health policies are often conducted at the local level, so, ideally, information is obtained at a fine spatial scale to facilitate interventions that can have the most significant impact [87]. Here, the ecological niche of schistosomiasis and human population density data were combined to produce a map of disease transmission risk. This method has been previously applied to research on the Zika virus (ZIKV) [76] and the fungal pathogen *Cryptococcus* [86] in Europe and the Americas.

The variables of significant contribution to the ecological niche model corroborate previous research reporting the significance of the distance to water [56], accessibility to healthcare resources [88,89], landscape characteristics [53,90,91], and temperature [92,93] as helpful in understanding the complex social-ecological systems associated with schistosomiasis. Long travel times are problematic in low-income settings because they are associated with increased travel costs and influence whether individuals seek critical care [61]. It is estimated that only 60–70% of the population of Madagascar has access to primary healthcare and that travel distances to primary care often exceed 10 km [94]. In 2014, Madagascar had the lowest reported healthcare spending globally (per capita) at $ 13.56 [95]. Equally relevant to the model, the distance to water represents the habitat for aquatic snail species and a foci of infection for humans when fishing, bathing, and swimming. Drivers of potential water contact patterns can extend outside of rivers, streams, and lakes. Sources vary from artificial irrigation canals, small reservoirs, and agricultural impoundments [96]. Water contact patterns and schistosomiasis transmission dynamics are additionally influenced by local cultural practices, socioeconomics, and spatiotemporal variability (i.e., seasonality) [96].

With the continued threat of climate change, the risk of neglected tropical diseases (NTDs) like schistosomiasis may increase, especially in low-resource communities [97]. The task of predicting the effects of climate change on schistosomiasis is complicated by the ecology of snails and parasite species and the scale of temperature and precipitation data [98,99]. Previous research has hypothesized that because of the host snails’ poikilotherm nature, changes in temperature and precipitation could alter reproduction, survival, and dispersal throughout the environment [100]. Currently, southern Madagascar is experiencing widespread severe drought, the worst in nearly 40 years. As a result, more than one million people are suffering from food insecurity and are on the brink of famine [101]. Some have speculated that this disaster is the first famine caused by the direct effects of climate change [102]. The ongoing COVID-19 global pandemic has posed several challenges for the mass treatment of schistosomiasis. The traditional MDA campaigns at schools have shifted to door-to-door campaigns, which have increased staff costs and are further complicated by poor road conditions throughout the country [32]. In addition, anthropogenic activities such as the construction of water development projects may create additional suitable habitats for the intermediate freshwater snail hosts; thus, allowing the risk of human infection to spread into previously nonendemic regions [103].

This study has some limitations. As previously stated, schistosomiasis is a focal disease strongly linked with the socioeconomic status of those infected. Contextual level factors like poverty, access to clean drinking water, and the safe disposal of human waste vary geographically. Therefore, the models presented here cannot extract causality or measure the disease’s prevalence or incidence. In addition, the risk to humans is complex and reflects personal, cultural habits, environmental factors, and the underlying societal structure. Thus, the estimated at-risk population should be viewed with caution as it does not reflect the real risk to the entire population (14.9 million). The study data was additionally extracted from historical and contemporary sources, so some uncertainties may be present, especially for the oldest records included in the analysis. More than likely, other endemic areas in Madagascar were not included in this study because records were not available.

To improve future mapping efforts, national geo-referenced survey data combined with macroecological information would be helpful. This would improve model accuracy and enable more precise interventions in priority areas. One limitation of the ENM model in this study is that it was developed with pseudoabsence data. Due to the lack of available true absence data, it was necessary to generate pseudoabsence data (*n* = 264). Previous research has documented the advantage of presence-absence techniques versus the random generation of pseudoabsences [104]. Although, even with this inherent methodological limitation, an ensemble model can produce a more robust prediction than a single model’s output [65].

## 5. Conclusions

In summary, this study mapped the environmental suitability and disease transmission risk of schistosomiasis in Madagascar for the first time. Significant findings from this research are as follows:

- The total area of environmental suitability is 264,781 km^2^ (102,232 sq miles).
- The population at risk is 14,972,194 million people (2020) (11.4 million-rural areas; 3.5 million-urban areas).
- Environmental suitability is concentrated throughout Sofia, Boeny, Bongolava, Itasy, Analamanga, Betsiboka, Alaotra-Mangoro, Atsinanana, Vakinankaratra, Amoron’I mania, Vatovavy Fitovinany, Haute Matsiatra, Menabe, Atsimo–Andrefana, Ihorombe, Anosy, Androy, and Atsimo-Atsinana.
- The disease transmission risk to human populations is significant within the central highland region, humid tropical eastern coast, dry-arid southwest, northwest, and to a lesser extent, the north and east.
- Variables of significance model contribution were the accessibility to cities, distance to water, enhanced vegetation index (EVI), annual mean temperature, land surface temperature (LST), clay content, and annual precipitation.

These maps can serve as a guideline for schistosomiasis control programs, which could prove beneficial to medical intervention campaigns. In addition, these maps can guide integrated disease surveillance and response systems in identifying schistosomiasis hot spots. Moreover, environmental-health education and targeted host snail-control programs can benefit from the risk maps presented here.

## Figures and Tables

**Table 1:** Environmental variables.

Variable	Included in Model	Source	Resolution	Unit	Average
BIO1–Annual Mean Temperature	Yes	WorldClim (v.2.1)	~1 km	°C	23.18
BIO2–Mean Diurnal Range	No	WorldClim (v.2.1)	~1 km	°C	11.93
BIO3–Isothermality	Yes	WorldClim (v.2.1)	~1 km	°C	65.13
BIO4–Temperature Seasonality	Yes	WorldClim (v.2.1)	~1 km	°C	232.32
BIO5–Max Temperature of Warmest Month	No	WorldClim (v.2.1)	~1 km	°C	31.46
BIO6–Min Temperature of Coldest Month	No	WorldClim (v.2.1)	~1 km	°C	13.12
BIO7–Temperature Annual Range		WorldClim (v.2.1)	~1 km	°C	18.33
BIO8–Mean Temperature of Wettest Quarter	No *	WorldClim (v.2.1)	~1 km	°C	-
BIO9–Mean Temperature of Driest Quarter	No *	WorldClim (v.2.1)	~1 km	°C	-
BIO10–Mean Temperature of Warmest Quarter	No	WorldClim (v.2.1)	~1 km	°C	25.49
BIO11–Mean Temperature of Coldest Quarter	No	WorldClim (v.2.1)	~1 km	°C	19.98
BIO12–Annual Precipitation	Yes	WorldClim (v.2.1)	~1 km	mm	1371.61
BIO13–Precipitation of Wettest Month	No	WorldClim (v.2.1)	~1 km	mm	310.46
BIO14–Precipitation of Driest Month	No	WorldClim (v.2.1)	~1 km	mm	18.59
BIO15–Precipitation Seasonality	Yes	WorldClim (v.2.1)	~1 km	mm	100.13
BIO16–Precipitation of Wettest Quarter	No	WorldClim (v.2.1)	~1 km	mm	808.67
BIO17–Precipitation of Driest Quarter	No	WorldClim (v.2.1)	~1 km	mm	68.88
BIO18–Precipitation of Warmest Quarter	No *	WorldClim (v.2.1)	~1 km	mm	-
BIO19–Precipitation of Coldest Quarter	No *	WorldClim (v.2.1)	~1 km	mm	-
Clay Content	Yes	SoilGrids	~1 km	g/100 g	23.68
Silt Content	Yes	SoilGrids	~1 km	g/100 g	15.84
Sand Content	No	SoilGrids	~1 km	g/100 g	60.80
Elevation	No	EarthEnv	~1 km	meters	465.11
Enhanced Vegetation Index (EVI)	Yes	WorldGrids	~1 km	0–6	2.96
Land Surface Temperature (LST)	Yes	WorldGrids	~1 km	°C	29.93
Distance to Water	Yes	DIV-GIS	~1 km	meters	2515.57
Accessibility to Cities	Yes	Malaria Atlas Project	~1 km	time	338.04
Nighttime Lights	Yes	NOAA	~1 km	1–63	5.97

* Excluded before modeling due to known spatial artifacts [58].

## Data Availability

Data is contained within the article or supplementary material. The data presented in this study are available in (see Supplementary Materials).

## Supplementary Materials

The following supporting information can be downloaded at: https://www.mdpi.com/article/10.3390/tropicalmed7020015/s1, Image S1: Variable response plots. Table S1: Occurrence locations in Madagascar (*n* = 231).
