# Supplementary material for: Predictive Risk Mapping of Schistosomiasis in Madagascar Using Ecological Niche Modeling and Precision Mapping
Source: Trop Med Infect Dis. 2022 Jan 19;7(2):15. doi: 10.3390/tropicalmed7020015 (PMC8876685; doi:10.3390/tropicalmed7020015)
Supplement: Supplementary file 1 [file tropicalmed-07-00015-s001.zip › tropicalmed-1518201-supplementary.pdf]

*Tropical Medicine and Infectious Disease*

## Supplementary Materials

*Article*

# Predictive Risk Mapping of Schistosomiasis in Madagascar Using Ecological Niche Modeling and Precision Mapping

**Mark A. Deka**

Centers for Disease Control and Prevention (CDC), 4770 Buford Hwy NE, Atlanta, GA 30341, USA; pmu5@cdc.gov

**Table of Contents**

*Image S1: Environmental variable response plots*

*Table S1: Occurrence locations (n = 231)*

**Image S1:** Environmental variable response plots.

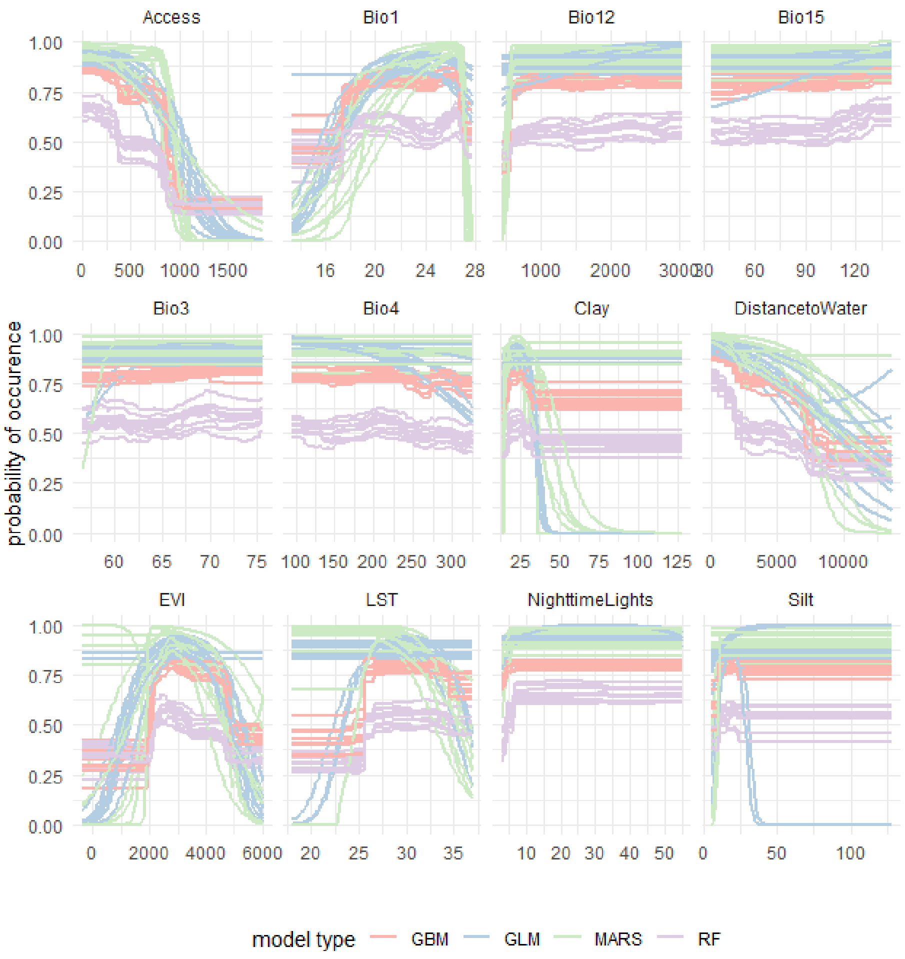

**Table S1.** Occurrence locations (n = 231).

| Source | Year | Species | Longitude | Latitude |
|---|---|---|---|---|
| **GBIF.org (11 November 2021) GBIF Occurrence Download https://doi.org/10.15468/dl.rmpbet.** | NA | *Bulinus forskalii* | 48.25899 | -13.31421 |
| **GBIF.org (11 November 2021) GBIF Occurrence Download https://doi.org/10.15468/dl.rmpbet.** | NA | *Bulinus forskalii* | 48.25899 | -13.31421 |
| **GBIF.org (11 November 2021) GBIF Occurrence Download https://doi.org/10.15468/dl.rmpbet.** | 1921 | *Bulinus forskalii* | 48.25899 | -13.31421 |
| **GBIF.org (11 November 2021) GBIF Occurrence Download https://doi.org/10.15468/dl.rmpbet.** | NA | *Bulinus forskalii* | 48.25899 | -13.31421 |
| **GBIF.org (11 November 2021) GBIF Occurrence Download https://doi.org/10.15468/dl.rmpbet.** | NA | *Bulinus forskalii* | 48.25899 | -13.31421 |
| **GBIF.org (11 November 2021) GBIF Occurrence Download https://doi.org/10.15468/dl.rmpbet.** | 1921 | *Bulinus forskalii* | 48.25899 | -13.31421 |
| **GBIF.org (11 November 2021) GBIF Occurrence Download https://doi.org/10.15468/dl.rmpbet.** | 1998 | *Bulinus liratus* | 48.753847 | -14.5473 |
| **GBIF.org (11 November 2021) GBIF Occurrence Download https://doi.org/10.15468/dl.rmpbet.** | 1998 | *Bulinus obtusispira* | 49.043632 | -13.193417 |
| **GBIF.org (11 November 2021) GBIF Occurrence Download https://doi.org/10.15468/dl.rmpbet.** | 1997 | *Bulinus bavayi* | 44.802203 | -23.560516 |
| **GBIF.org (11 November 2021) GBIF Occurrence Download https://doi.org/10.15468/dl.rmpbet.** | 1998 | *Bulinus truncatus* | 44.713 | -24.665417 |
| **GBIF.org (11 November 2021) GBIF Occurrence Download https://doi.org/10.15468/dl.rmpbet.** | 1997 | *Bulinus truncatus* | 48.660183 | -14.66555 |
| **GBIF.org (11 November 2021) GBIF Occurrence Download https://doi.org/10.15468/dl.rmpbet.** | 1997 | *Bulinus liratus* | 44.802203 | -23.560516 |
| **GBIF.org (11 November 2021) GBIF Occurrence Download https://doi.org/10.15468/dl.rmpbet.** | 2008 | *Bulinus africanus* | 47.33252 | -23.20214 |
| **GBIF.org (11 November 2021) GBIF Occurrence Download https://doi.org/10.15468/dl.rmpbet.** | 1997 | *Bulinus liratus* | 44.520767 | -23.4906 |
| **GBIF.org (11 November 2021) GBIF Occurrence Download https://doi.org/10.15468/dl.rmpbet.** | 1997 | *Bulinus truncatus* | 44.520767 | -23.4906 |
| **GBIF.org (11 November 2021) GBIF Occurrence Download https://doi.org/10.15468/dl.rmpbet.** | 2008 | *Bulinus truncatus* | 45.66405 | -24.2064 |
| **GBIF.org (11 November 2021) GBIF Occurrence Download https://doi.org/10.15468/dl.ntsbnv.** | NA | *Biomphalaria pfeifferi* | 47 | -20 |
| **GBIF.org (11 November 2021) GBIF Occurrence Download https://doi.org/10.15468/dl.ntsbnv.** | NA | *Biomphalaria pfeifferi* | 48.403435 | -14.899761 |
| **Clinical findings in female genital schistosomiasis in Madagascar** | 1997 | *S. haematobium* | 45.483002 | -19.615 |
| **Ultrasonographical assessment of morbidity in schistosomiasis mansoni in Madagascar: a community-based study in a rural population** | 1993 | *S. mansoni* | 45.932999 | -19.082001 |
| **Ultrasonographical assessment of morbidity in schistosomiasis mansoni in Madagascar: a community-based study in a rural population** | 1993 | *S. mansoni* | 45.812 | -18.905001 |

| | | | | |
|---|---|---|---|---|
| **Bilan parasitologique d'une population du nord ouest de Madagascar avant travaux d'amenagement hydraulique pour l'extension d'un complexe sucrier (Ambilobe). / Parasitological status of a population of north-western Madagascar before the construction of** | 1981 | *S. haematobium* | 48.849998 | -13.083 |
| **Bilan parasitologique d'une population du nord ouest de Madagascar avant travaux d'amenagement hydraulique pour l'extension d'un complexe sucrier (Ambilobe). / Parasitological status of a population of north-western Madagascar before the construction of** | 1981 | *S. haematobium* | 49.050999 | -13.194 |
| **Bilan parasitologique d'une population du nord ouest de Madagascar avant travaux d'amenagement hydraulique pour l'extension d'un complexe sucrier (Ambilobe). / Parasitological status of a population of north-western Madagascar before the construction of** | 1981 | *S. haematobium* | 48.866001 | -13.335 |
| **Bilan parasitologique d'une population du nord ouest de Madagascar avant travaux d'amenagement hydraulique pour l'extension d'un complexe sucrier (Ambilobe). / Parasitological status of a population of north-western Madagascar before the construction of** | 1981 | *S. haematobium* | 48.882999 | -13.116 |
| **Sexual behavior and sexually transmitted infections in men living in rural Madagascar: implications for HIV transmission** | 1998 | *S. haematobium* | 46.098999 | -22.365999 |
| **Sexual behavior and sexually transmitted infections in men living in rural Madagascar: implications for HIV transmission** | 1998 | *S. haematobium* | 43.6833 | -23.3167 |
| **Sero-epidemiological study of intestinal schistosomiasis at Ampefy (Itasy Lake, Madagascar)** | 1983 | *S. mansoni* | 46.733299 | -19.049999 |
| **Study of schistosomiasis in the Lake Aloatra region - the "rice granary" of Madagascar** | 1982 | *S. mansoni* | 48.398998 | -17.9 |
| **Study of schistosomiasis in the Lake Aloatra region - the "rice granary" of Madagascar** | 1982 | *S. mansoni* | 48.495998 | -17.406 |
| **Study of schistosomiasis in the Lake Aloatra region - the "rice granary" of Madagascar** | 1982 | *S. mansoni* | 48.516701 | -17.616699 |
| **Study of schistosomiasis in the Lake Aloatra region - the "rice granary" of Madagascar** | 1982 | *S. mansoni* | 48.5 | -17.632999 |
| **Study of schistosomiasis in the Lake Aloatra region - the "rice granary" of Madagascar** | 1982 | *S. mansoni* | 48.432999 | -17.750999 |
| **Study of schistosomiasis in the Lake Aloatra region - the "rice granary" of Madagascar** | 1982 | *S. mansoni* | 48.633301 | -17.3333 |
| **Study of schistosomiasis in the Lake Aloatra region - the "rice granary" of Madagascar** | 1982 | *S. mansoni* | 48.429001 | -17.514999 |
| **Schistosoma mansoni in schoolchildren in a Madagascan highland school assessed by PCR and sedimentation microscopy and Bayesian estimation of sensitivities and specificities** | 2012 | *S. mansoni* | 47.155 | -20.5235 |
| **Schistosoma mansoni in schoolchildren in a Madagascan highland school assessed by PCR and sedimentation microscopy and Bayesian estimation of sensitivities and specificities** | 2012 | *S. mansoni* | 47.155 | -20.5235 |

| | | | | |
|---|---|---|---|---|
| **Parasitic co-infections: does Ascaris lumbricoides protect against Plasmodium falciparum infection?** | 1997 | *S. mansoni* | 46.549999 | -19.383301 |
| **Morbidity of schistosomiasis mansoni in the highlands of Madagascar and comparison of current sonographical classification systems** | 1994 | *S. mansoni* | 46.833 | -20.549 |
| **Worms, wells, and water in western Madagascar** | 1987 | *S. haematobium* | 44.632 | -20.282 |
| **Lot quality assurance sampling for screening communities hyperendemic for Schistosoma mansoni** | 1998 | *S. mansoni* | 46.316002 | -22.233 |
| **Lot quality assurance sampling for screening communities hyperendemic for Schistosoma mansoni** | 1998 | *S. mansoni* | 46.082001 | -22.333 |
| **Lot quality assurance sampling for screening communities hyperendemic for Schistosoma mansoni** | 1998 | *S. mansoni* | 46.366001 | -22.049999 |
| **Lot quality assurance sampling for screening communities hyperendemic for Schistosoma mansoni** | 1998 | *S. mansoni* | 45.799 | -22.615999 |
| **Lot quality assurance sampling for screening communities hyperendemic for Schistosoma mansoni** | 1998 | *S. mansoni* | 46.083302 | -22.35 |
| **Lot quality assurance sampling for screening communities hyperendemic for Schistosoma mansoni** | 1998 | *S. mansoni* | 46.231998 | -22.316 |
| **Lot quality assurance sampling for screening communities hyperendemic for Schistosoma mansoni** | 1998 | *S. mansoni* | 46.4333 | -23.450001 |
| **Lot quality assurance sampling for screening communities hyperendemic for Schistosoma mansoni** | 1998 | *S. mansoni* | 46.483002 | -22.6 |
| **Lot quality assurance sampling for screening communities hyperendemic for Schistosoma mansoni** | 1998 | *S. mansoni* | 46.283001 | -22.499001 |
| **Lot quality assurance sampling for screening communities hyperendemic for Schistosoma mansoni** | 1998 | *S. mansoni* | 46.368 | -22.573 |
| **Lot quality assurance sampling for screening communities hyperendemic for Schistosoma mansoni** | 1998 | *S. mansoni* | 46.115002 | -22.450001 |
| **Lot quality assurance sampling for screening communities hyperendemic for Schistosoma mansoni** | 1998 | *S. mansoni* | 45.6833 | -22.0833 |
| **Lot quality assurance sampling for screening communities hyperendemic for Schistosoma mansoni** | 1998 | *S. mansoni* | 46.583 | -22.483999 |
| **Lot quality assurance sampling for screening communities hyperendemic for Schistosoma mansoni** | 1998 | *S. mansoni* | 46.368 | -22.211 |
| **Lot quality assurance sampling for screening communities hyperendemic for Schistosoma mansoni** | 1998 | *S. mansoni* | 45.75 | -22.516001 |
| **Lot quality assurance sampling for screening communities hyperendemic for Schistosoma mansoni** | 1998 | *S. mansoni* | 46.166 | -22.35 |
| **Lot quality assurance sampling for screening communities hyperendemic for Schistosoma mansoni** | 1998 | *S. mansoni* | 46.098999 | -22.431999 |
| **Lot quality assurance sampling for screening communities hyperendemic for Schistosoma mansoni** | 1998 | *S. mansoni* | 46.150002 | -22.316 |
| **Lot quality assurance sampling for screening communities hyperendemic for Schistosoma mansoni** | 1998 | *S. mansoni* | 46.098999 | -22.431999 |
| **Lot quality assurance sampling for screening communities hyperendemic for Schistosoma mansoni** | 1998 | *S. mansoni* | 46.150002 | -22.316 |
| **Lot quality assurance sampling for screening communities hyperendemic for Schistosoma mansoni** | 1998 | *S. mansoni* | 45.75 | -22.516001 |

| | | | | |
|---|---|---|---|---|
| **Lot quality assurance sampling for screening communities hyperendemic for Schistosoma mansoni** | 1998 | *S. mansoni* | 46.150002 | -22.466 |
| **Lot quality assurance sampling for screening communities hyperendemic for Schistosoma mansoni** | 1998 | *S. mansoni* | 45.999001 | -22.433001 |
| **Lot quality assurance sampling for screening communities hyperendemic for Schistosoma mansoni** | 1998 | *S. mansoni* | 46.499001 | -22.632999 |
| **Lot quality assurance sampling for screening communities hyperendemic for Schistosoma mansoni** | 1998 | *S. mansoni* | 46.098999 | -22.367001 |
| **Lot quality assurance sampling for screening communities hyperendemic for Schistosoma mansoni** | 1998 | *S. mansoni* | 45.999001 | -22.532 |
| **Lot quality assurance sampling for screening communities hyperendemic for Schistosoma mansoni** | 1998 | *S. mansoni* | 46.150002 | -22.466 |
| **Lot quality assurance sampling for screening communities hyperendemic for Schistosoma mansoni** | 1998 | *S. mansoni* | 46.499001 | -22.533001 |
| **Lot quality assurance sampling for screening communities hyperendemic for Schistosoma mansoni** | 1998 | *S. mansoni* | 45.733299 | -22.6833 |
| **Lot quality assurance sampling for screening communities hyperendemic for Schistosoma mansoni** | 1998 | *S. mansoni* | 46.116699 | -22.75 |
| **Lot quality assurance sampling for screening communities hyperendemic for Schistosoma mansoni** | 1998 | *S. mansoni* | 46.283298 | -22.483299 |
| **Lot quality assurance sampling for screening communities hyperendemic for Schistosoma mansoni** | 1998 | *S. mansoni* | 46.098999 | -22.4 |
| **Ultrasonographical assessment of morbidity in Schistosoma mansoni infections in Madagascar according to two classifications: Cairo/WHO and Managil-Hannover staging-systems** | 1996 | *S. mansoni* | 46.733299 | -19.049999 |
| **Ultrasonographical assessment of morbidity in Schistosoma mansoni infections in Madagascar according to two classifications: Cairo/WHO and Managil-Hannover staging-systems** | 1996 | *S. mansoni* | 46.782002 | -18.85 |
| **Potential spread of Schistosoma mansoni in Antananarivo and suburbs according to present epidemiological data** | 1994 | *S. mansoni* | 47.083 | -19.365999 |
| **Potential spread of Schistosoma mansoni in Antananarivo and suburbs according to present epidemiological data** | 1994 | *S. mansoni* | 47.015999 | -17.916 |
| **Potential spread of Schistosoma mansoni in Antananarivo and suburbs according to present epidemiological data** | 1994 | *S. mansoni* | 47.5 | -18.931999 |
| **Potential spread of Schistosoma mansoni in Antananarivo and suburbs according to present epidemiological data** | 1994 | *S. mansoni* | 47.5 | -18.931999 |
| **Potential spread of Schistosoma mansoni in Antananarivo and suburbs according to present epidemiological data** | 1994 | *S. mansoni* | 47.566002 | -18.948 |
| **Potential spread of Schistosoma mansoni in Antananarivo and suburbs according to present epidemiological data** | 1994 | *S. mansoni* | 47.083 | -18.283001 |

| | | | | |
|---|---|---|---|---|
| **Potential spread of Schistosoma mansoni in Antananarivo and suburbs according to present epidemiological data** | 1994 | *S. mansoni* | 46.849998 | -18.8333 |
| **Potential spread of Schistosoma mansoni in Antananarivo and suburbs according to present epidemiological data** | 1994 | *S. mansoni* | 47.015999 | -17.916 |
| **Potential spread of Schistosoma mansoni in Antananarivo and suburbs according to present epidemiological data** | 1994 | *S. mansoni* | 47.5 | -18.716 |
| **Potential spread of Schistosoma mansoni in Antananarivo and suburbs according to present epidemiological data** | 1994 | *S. mansoni* | 47.533001 | -18.931999 |
| **Potential spread of Schistosoma mansoni in Antananarivo and suburbs according to present epidemiological data** | 1994 | *S. mansoni* | 47.533001 | -18.931999 |
| **Potential spread of Schistosoma mansoni in Antananarivo and suburbs according to present epidemiological data** | 1994 | *S. mansoni* | 47.5 | -18.899 |
| **Potential spread of Schistosoma mansoni in Antananarivo and suburbs according to present epidemiological data** | 1994 | *S. haematobium* | 46.849998 | -18.8333 |
| **Potential spread of Schistosoma mansoni in Antananarivo and suburbs according to present epidemiological data** | 1994 | *S. mansoni* | 46.849998 | -18.8333 |
| **Potential spread of Schistosoma mansoni in Antananarivo and suburbs according to present epidemiological data** | 1994 | *S. mansoni* | 47.566002 | -18.999001 |
| **Potential spread of Schistosoma mansoni in Antananarivo and suburbs according to present epidemiological data** | 1994 | *S. mansoni* | 47.483002 | -18.216 |
| **Potential spread of Schistosoma mansoni in Antananarivo and suburbs according to present epidemiological data** | 1994 | *S. mansoni* | 47.549 | -18.85 |
| **Potential spread of Schistosoma mansoni in Antananarivo and suburbs according to present epidemiological data** | 1994 | *S. mansoni* | 47.533901 | -18.8904 |
| **Potential spread of Schistosoma mansoni in Antananarivo and suburbs according to present epidemiological data** | 1994 | *S. mansoni* | 47.533001 | -18.833 |
| **Reagent strips: their interest for Schistosoma haematobium mass screening in Madagascar** | 1994 | *S. haematobium* | 45.483002 | -19.615 |
| **Schistosoma haematobium infection in western Madagascar: morbidity determined by ultrasonography** | 1994 | *S. haematobium* | 45.483002 | -19.615 |
| **Geographic differences in hepatosplenic complications of schistosomiasis mansoni and explanatory factors of morbidity** | 1997 | *S. mansoni* | 46.283298 | -22.483299 |
| **Geographic differences in hepatosplenic complications of schistosomiasis mansoni and explanatory factors of morbidity** | 1997 | *S. mansoni* | 46.368 | -22.211 |

| | | | | |
|---|---|---|---|---|
| **Geographic differences in hepatosplenic complications of schistosomiasis mansoni and explanatory factors of morbidity** | 1997 | *S. mansoni* | 46.366001 | -22.049999 |
| **Identification of communities endemic for urinary bilharziosis by the "Lot Quality Assurance Sampling" method in Madagascar** | 2000 | *S. haematobium* | 46.700001 | -16.166 |
| **Identification of communities endemic for urinary bilharziosis by the "Lot Quality Assurance Sampling" method in Madagascar** | 2000 | *S. haematobium* | 45.592999 | -20.349001 |
| **Identification of communities endemic for urinary bilharziosis by the "Lot Quality Assurance Sampling" method in Madagascar** | 2000 | *S. haematobium* | 45.592999 | -20.349001 |
| **Identification of communities endemic for urinary bilharziosis by the "Lot Quality Assurance Sampling" method in Madagascar** | 2000 | *S. haematobium* | 45.592999 | -20.349001 |
| **Validation of questionnaire methods to identify Schistosoma haematobium bilharziasis hyperendemic zones in Madagascar** | 1999 | *S. haematobium* | 43.659 | -22.179001 |
| **Validation of questionnaire methods to identify Schistosoma haematobium bilharziasis hyperendemic zones in Madagascar** | 1999 | *S. haematobium* | 44.535999 | -24.813 |
| **Validation of questionnaire methods to identify Schistosoma haematobium bilharziasis hyperendemic zones in Madagascar** | 1999 | *S. haematobium* | 44.816002 | -23.716 |
| **Validation of questionnaire methods to identify Schistosoma haematobium bilharziasis hyperendemic zones in Madagascar** | 1999 | *S. haematobium* | 44.816002 | -24.499001 |
| **Validation of questionnaire methods to identify Schistosoma haematobium bilharziasis hyperendemic zones in Madagascar** | 1999 | *S. haematobium* | 45.165001 | -25.183001 |
| **Validation of questionnaire methods to identify Schistosoma haematobium bilharziasis hyperendemic zones in Madagascar** | 1999 | *S. haematobium* | 44.744999 | -24.698999 |
| **Validation of questionnaire methods to identify Schistosoma haematobium bilharziasis hyperendemic zones in Madagascar** | 1999 | *S. haematobium* | 44.400002 | -20.1667 |
| **Validation of questionnaire methods to identify Schistosoma haematobium bilharziasis hyperendemic zones in Madagascar** | 1999 | *S. haematobium* | 44.4333 | -20.1833 |
| **Validation of questionnaire methods to identify Schistosoma haematobium bilharziasis hyperendemic zones in Madagascar** | 1999 | *S. haematobium* | 44.764999 | -24.65 |
| **Validation of questionnaire methods to identify Schistosoma haematobium bilharziasis hyperendemic zones in Madagascar** | 1999 | *S. haematobium* | 43.616699 | -22.25 |
| **Validation of questionnaire methods to identify Schistosoma haematobium bilharziasis hyperendemic zones in Madagascar** | 1999 | *S. haematobium* | 44.0667 | -23.733299 |

| | | | | |
|---|---|---|---|---|
| **Validation of questionnaire methods to identify Schistosoma haematobium bilharziasis hyperendemic zones in Madagascar** | 1999 | *S. haematobium* | 43.681999 | -22.282 |
| **Validation of questionnaire methods to identify Schistosoma haematobium bilharziasis hyperendemic zones in Madagascar** | 1999 | *S. haematobium* | 43.833302 | -22.1667 |
| **WHO. Atlas of the Global Distribution of Schistosomiasis - Madagascar - Mauritius. (1987)** | 1956 | *S. mansoni* | 49.396678 | -18.16201 |
| **WHO. Atlas of the Global Distribution of Schistosomiasis - Madagascar - Mauritius. (1987)** | 1971 | *S. haematobium* | 46.3261 | -15.705726 |
| **WHO. Atlas of the Global Distribution of Schistosomiasis - Madagascar - Mauritius. (1987)** | 1956 | *S. haematobium* | 46.692229 | -15.481616 |
| **WHO. Atlas of the Global Distribution of Schistosomiasis - Madagascar - Mauritius. (1987)** | 1956 | *S. haematobium* | 46.240692 | -15.762139 |
| **WHO. Atlas of the Global Distribution of Schistosomiasis - Madagascar - Mauritius. (1987)** | 1956 | *S. haematobium* | 44.867616 | -16.348874 |
| **WHO. Atlas of the Global Distribution of Schistosomiasis - Madagascar - Mauritius. (1987)** | 1956 | *S. haematobium* | 44.485813 | -16.743123 |
| **WHO. Atlas of the Global Distribution of Schistosomiasis - Madagascar - Mauritius. (1987)** | 1956 | *S. haematobium* | 45.103445 | -16.971656 |
| **WHO. Atlas of the Global Distribution of Schistosomiasis - Madagascar - Mauritius. (1987)** | 1956 | *S. haematobium* | 43.95664 | -17.51135 |
| **WHO. Atlas of the Global Distribution of Schistosomiasis - Madagascar - Mauritius. (1987)** | 1957 | *S. haematobium* | 44.030845 | -18.056023 |
| **WHO. Atlas of the Global Distribution of Schistosomiasis - Madagascar - Mauritius. (1987)** | 1956 | *S. haematobium* | 44.316126 | -18.566662 |
| **WHO. Atlas of the Global Distribution of Schistosomiasis - Madagascar - Mauritius. (1987)** | 1956 | *S. haematobium* | 44.342686 | -19.026085 |
| **WHO. Atlas of the Global Distribution of Schistosomiasis - Madagascar - Mauritius. (1987)** | 1956 | *S. haematobium* | 44.927724 | -17.821386 |
| **WHO. Atlas of the Global Distribution of Schistosomiasis - Madagascar - Mauritius. (1987)** | 1957 | *S. haematobium* | 45.256023 | -18.546364 |
| **WHO. Atlas of the Global Distribution of Schistosomiasis - Madagascar - Mauritius. (1987)** | 1952 | *S. haematobium* | 44.62393 | -18.673742 |
| **WHO. Atlas of the Global Distribution of Schistosomiasis - Madagascar - Mauritius. (1987)** | 1973 | *S. haematobium* | 44.54296 | -19.699319 |
| **WHO. Atlas of the Global Distribution of Schistosomiasis - Madagascar - Mauritius. (1987)** | 1956 | *S. haematobium* | 44.688781 | -19.705983 |
| **WHO. Atlas of the Global Distribution of Schistosomiasis - Madagascar - Mauritius. (1987)** | 1950 | *S. haematobium* | 44.963282 | -19.72856 |
| **WHO. Atlas of the Global Distribution of Schistosomiasis - Madagascar - Mauritius. (1987)** | 1956 | *S. haematobium* | 44.503774 | -20.328885 |
| **WHO. Atlas of the Global Distribution of Schistosomiasis - Madagascar - Mauritius. (1987)** | 1961 | *S. haematobium* | 44.360603 | -20.577678 |
| **WHO. Atlas of the Global Distribution of Schistosomiasis - Madagascar - Mauritius. (1987)** | 1956 | *S. haematobium* | 45.052792 | -20.411643 |
| **WHO. Atlas of the Global Distribution of Schistosomiasis - Madagascar - Mauritius. (1987)** | 1956 | *S. haematobium* | 44.929036 | -21.051182 |
| **WHO. Atlas of the Global Distribution of Schistosomiasis - Madagascar - Mauritius. (1987)** | 1952 | *S. haematobium* | 43.601274 | -21.322382 |

| | | | | |
|---|---|---|---|---|
| **WHO. Atlas of the Global Distribution of Schistosomiasis - Madagascar - Mauritius. (1987)** | 1957 | *S. haematobium* | 43.889364 | -21.714482 |
| **WHO. Atlas of the Global Distribution of Schistosomiasis - Madagascar - Mauritius. (1987)** | 1956 | *S. haematobium* | 45.065014 | -22.172659 |
| **WHO. Atlas of the Global Distribution of Schistosomiasis - Madagascar - Mauritius. (1987)** | 1957 | *S. haematobium* | 43.761056 | -23.547822 |
| **WHO. Atlas of the Global Distribution of Schistosomiasis - Madagascar - Mauritius. (1987)** | 1950 | *S. haematobium* | 44.681999 | -22.650675 |
| **WHO. Atlas of the Global Distribution of Schistosomiasis - Madagascar - Mauritius. (1987)** | 1951 | *S. haematobium* | 48.084039 | -14.204835 |
| **WHO. Atlas of the Global Distribution of Schistosomiasis - Madagascar - Mauritius. (1987)** | 1973 | *S. haematobium* | 49.64809 | -14.655774 |
| **WHO. Atlas of the Global Distribution of Schistosomiasis - Madagascar - Mauritius. (1987)** | 1965 | *S. haematobium* | 49.947364 | -13.860145 |
| **WHO. Atlas of the Global Distribution of Schistosomiasis - Madagascar - Mauritius. (1987)** | 1965 | *S. haematobium* | 49.661299 | -13.20527 |
| **WHO. Atlas of the Global Distribution of Schistosomiasis - Madagascar - Mauritius. (1987)** | 1976 | *S. haematobium* | 49.234433 | -13.156635 |
| **WHO. Atlas of the Global Distribution of Schistosomiasis - Madagascar - Mauritius. (1987)** | 1955 | *S. haematobium* | 49.015587 | -13.047098 |
| **WHO. Atlas of the Global Distribution of Schistosomiasis - Madagascar - Mauritius. (1987)** | 1958 | *S. haematobium* | 48.274596 | -13.39859 |
| **WHO. Atlas of the Global Distribution of Schistosomiasis - Madagascar - Mauritius. (1987)** | 1950 | *S. haematobium* | 47.395576 | -15.121899 |
| **WHO. Atlas of the Global Distribution of Schistosomiasis - Madagascar - Mauritius. (1987)** | 1952 | *S. haematobium* | 47.738956 | -14.605437 |
| **WHO. Atlas of the Global Distribution of Schistosomiasis - Madagascar - Mauritius. (1987)** | 1965 | *S. haematobium* | 47.711414 | -14.405327 |
| **WHO. Atlas of the Global Distribution of Schistosomiasis - Madagascar - Mauritius. (1987)** | 1976 | *S. haematobium* | 48.823157 | -13.550206 |
| **WHO. Atlas of the Global Distribution of Schistosomiasis - Madagascar - Mauritius. (1987)** | 1956 | *S. haematobium* | 48.750488 | -14.542363 |
| **WHO. Atlas of the Global Distribution of Schistosomiasis - Madagascar - Mauritius. (1987)** | 1957 | *S. haematobium* | 48.54612 | -15.041382 |
| **WHO. Atlas of the Global Distribution of Schistosomiasis - Madagascar - Mauritius. (1987)** | 1956 | *S. haematobium* | 48.535774 | -15.741076 |
| **WHO. Atlas of the Global Distribution of Schistosomiasis - Madagascar - Mauritius. (1987)** | 1956 | *S. haematobium* | 47.735283 | -15.934238 |
| **WHO. Atlas of the Global Distribution of Schistosomiasis - Madagascar - Mauritius. (1987)** | 1958 | *S. haematobium* | 47.992812 | -14.882714 |
| **WHO. Atlas of the Global Distribution of Schistosomiasis - Madagascar - Mauritius. (1987)** | 1956 | *S. haematobium* | 47.918562 | -15.544481 |
| **WHO. Atlas of the Global Distribution of Schistosomiasis - Madagascar - Mauritius. (1987)** | 1956 | *S. haematobium* | 46.956888 | -17.305317 |
| **WHO. Atlas of the Global Distribution of Schistosomiasis - Madagascar - Mauritius. (1987)** | 1956 | *S. haematobium* | 47.651022 | -16.797774 |
| **WHO. Atlas of the Global Distribution of Schistosomiasis - Madagascar - Mauritius. (1987)** | 1954 | *S. mansoni* | 49.454667 | -17.489337 |
| **WHO. Atlas of the Global Distribution of Schistosomiasis - Madagascar - Mauritius. (1987)** | 1957 | *S. mansoni* | 49.139175 | -17.432439 |

| | | | | |
|---|---|---|---|---|
| **WHO. Atlas of the Global Distribution of Schistosomiasis - Madagascar - Mauritius. (1987)** | 1956 | *S. mansoni* | 46.812301 | -25.027343 |
| **WHO. Atlas of the Global Distribution of Schistosomiasis - Madagascar - Mauritius. (1987)** | 1955 | *S. mansoni* | 46.687384 | -25.05382 |
| **WHO. Atlas of the Global Distribution of Schistosomiasis - Madagascar - Mauritius. (1987)** | 1955 | *S. mansoni* | 46.965085 | -24.569246 |
| **WHO. Atlas of the Global Distribution of Schistosomiasis - Madagascar - Mauritius. (1987)** | 1955 | *S. mansoni* | 47.547207 | -23.798909 |
| **WHO. Atlas of the Global Distribution of Schistosomiasis - Madagascar - Mauritius. (1987)** | 1954 | *S. mansoni* | 46.971859 | -24.020175 |
| **WHO. Atlas of the Global Distribution of Schistosomiasis - Madagascar - Mauritius. (1987)** | 1971 | *S. mansoni* | 47.597946 | -23.339606 |
| **WHO. Atlas of the Global Distribution of Schistosomiasis - Madagascar - Mauritius. (1987)** | 1955 | *S. mansoni* | 45.061314 | -25.174255 |
| **WHO. Atlas of the Global Distribution of Schistosomiasis - Madagascar - Mauritius. (1987)** | 1950 | *S. mansoni* | 46.079448 | -24.072031 |
| **WHO. Atlas of the Global Distribution of Schistosomiasis - Madagascar - Mauritius. (1987)** | 1955 | *S. mansoni* | 46.476443 | -24.604196 |
| **WHO. Atlas of the Global Distribution of Schistosomiasis - Madagascar - Mauritius. (1987)** | 1955 | *S. mansoni* | 45.595353 | -24.673199 |
| **WHO. Atlas of the Global Distribution of Schistosomiasis - Madagascar - Mauritius. (1987)** | 1955 | *S. mansoni* | 45.302332 | -24.558602 |
| **WHO. Atlas of the Global Distribution of Schistosomiasis - Madagascar - Mauritius. (1987)** | 1975 | *S. mansoni* | 44.362292 | -23.818446 |
| **WHO. Atlas of the Global Distribution of Schistosomiasis - Madagascar - Mauritius. (1987)** | 1955 | *S. mansoni* | 46.091328 | -25.17356 |
| **WHO. Atlas of the Global Distribution of Schistosomiasis - Madagascar - Mauritius. (1987)** | 1953 | *S. mansoni* | 45.012415 | -23.545244 |
| **WHO. Atlas of the Global Distribution of Schistosomiasis - Madagascar - Mauritius. (1987)** | 1968 | *S. mansoni* | 49.001243 | -18.470049 |
| **WHO. Atlas of the Global Distribution of Schistosomiasis - Madagascar - Mauritius. (1987)** | 1961 | *S. mansoni* | 48.976676 | -19.330777 |
| **WHO. Atlas of the Global Distribution of Schistosomiasis - Madagascar - Mauritius. (1987)** | 1961 | *S. mansoni* | 48.80693 | -19.893801 |
| **WHO. Atlas of the Global Distribution of Schistosomiasis - Madagascar - Mauritius. (1987)** | 1959 | *S. mansoni* | 49.415774 | -17.660979 |
| **WHO. Atlas of the Global Distribution of Schistosomiasis - Madagascar - Mauritius. (1987)** | 1957 | *S. mansoni* | 47.034079 | -22.500241 |
| **WHO. Atlas of the Global Distribution of Schistosomiasis - Madagascar - Mauritius. (1987)** | 1954 | *S. mansoni* | 48.147637 | -21.229374 |
| **WHO. Atlas of the Global Distribution of Schistosomiasis - Madagascar - Mauritius. (1987)** | 1954 | *S. mansoni* | 48.345482 | -21.23837 |
| **WHO. Atlas of the Global Distribution of Schistosomiasis - Madagascar - Mauritius. (1987)** | 1953 | *S. mansoni* | 48.479514 | -20.773606 |
| **WHO. Atlas of the Global Distribution of Schistosomiasis - Madagascar - Mauritius. (1987)** | 1957 | *S. mansoni* | 48.600427 | -18.900622 |
| **WHO. Atlas of the Global Distribution of Schistosomiasis - Madagascar - Mauritius. (1987)** | 1951 | *S. mansoni* | 47.400828 | -20.307152 |
| **WHO. Atlas of the Global Distribution of Schistosomiasis - Madagascar - Mauritius. (1987)** | 1935 | *S. mansoni* | 48.322593 | -20.597505 |

| | | | | |
|---|---|---|---|---|
| **WHO. Atlas of the Global Distribution of Schistosomiasis - Madagascar - Mauritius. (1987)** | 1955 | *S. mansoni* | 47.041834 | -21.556506 |
| **WHO. Atlas of the Global Distribution of Schistosomiasis - Madagascar - Mauritius. (1987)** | 1955 | *S. mansoni* | 44.635148 | -22.090054 |
| **WHO. Atlas of the Global Distribution of Schistosomiasis - Madagascar - Mauritius. (1987)** | 1955 | *S. mansoni* | 44.857337 | -21.37432 |
| **WHO. Atlas of the Global Distribution of Schistosomiasis - Madagascar - Mauritius. (1987)** | 1955 | *S. mansoni* | 45.954758 | -20.532126 |
| **WHO. Atlas of the Global Distribution of Schistosomiasis - Madagascar - Mauritius. (1987)** | 1956 | *S. mansoni* | 47.553352 | -17.034229 |
| **WHO. Atlas of the Global Distribution of Schistosomiasis - Madagascar - Mauritius. (1987)** | 1957 | *S. mansoni* | 48.802676 | -19.57594 |
| **WHO. Atlas of the Global Distribution of Schistosomiasis - Madagascar - Mauritius. (1987)** | 1956 | *S. mansoni* | 46.914577 | -20.380735 |
| **High burden of Schistosoma mansoni infection in school-aged children in Marolambo District, Madagascar** | 2017 | Human Case | 48.099899 | -20.116435 |
| **High burden of Schistosoma mansoni infection in school-aged children in Marolambo District, Madagascar** | 2017 | Human Case | 47.940974 | -20.578717 |
| **Serology- and Blood-PCR-Based Screening for Schistosomiasis in Pregnant Women in Madagascar—A Cross-Sectional Study and Test Comparison Approach** | 2021 | Human Case | 46.049136 | -18.76977 |
| **Sawyer, S. G. (2013). Schistosomiasis (Bilharzia) in Madagascar: A Case Study of a Neglected Tropical Disease.** | 2013 | Human Case | 44.279857 | -20.294073 |
| **Usefulness of environmental DNA for detecting Schistosoma mansoni occurrence sites in Madagascar** | 2018 | Human Case | 46.964915 | -17.453877 |
| **Clinical findings in female genital schistosomiasis in Madagascar** | 1998 | Human Case | 44.459261 | -21.70056 |
| **Semen quality in Schistosoma haematobium infected men in Madagascar** | 2009 | Human Case | 48.928219 | -13.090937 |
| **The first cases of bovine schistosomiasis in Madagascar** | 1972 | Animal Case | 47.103564 | -21.452462 |
| **Gynecological Manifestations, Histopathological Findings, and Schistosoma-Specific Polymerase Chain Reaction Results Among Women with Schistosoma haematobium Infection: A Cross-sectional Study in Madagascar** | 2015 | Human Case | 45.456016 | -19.529578 |
| **Sexual Behavior and Sexually Transmitted Infections in Men Living in Rural Madagascar** | 2003 | Human Case | 47.035557 | -19.847871 |
| **Sexual Behavior and Sexually Transmitted Infections in Men Living in Rural Madagascar** | 2003 | Human Case | 46.09153 | -22.374414 |
| **An Isolated Focus of Intestinal Schistosomiasis in Madagascar.** | 1952 | Human Case | 48.835307 | -15.850427 |
| **An Isolated Focus of Intestinal Schistosomiasis in Madagascar.** | 1952 | Human Case | 47.198984 | -19.415002 |
| **WHO. Atlas of the Global Distribution of Schistosomiasis - Madagascar - Mauritius. (1987)** | 1971 | *S. mansoni* | 47.527049 | -23.267175 |
| **WHO. Atlas of the Global Distribution of Schistosomiasis - Madagascar - Mauritius. (1987)** | 1953 | *S. mansoni* | 47.317439 | -23.482813 |

| | | | | |
|---|---|---|---|---|
| **WHO. Atlas of the Global Distribution of Schistosomiasis - Madagascar - Mauritius. (1987)** | 1957 | *S. mansoni* | 46.957956 | -22.711437 |
| **WHO. Atlas of the Global Distribution of Schistosomiasis - Madagascar - Mauritius. (1987)** | 1953 | *S. mansoni* | 47.294772 | -22.657318 |
| **WHO. Atlas of the Global Distribution of Schistosomiasis - Madagascar - Mauritius. (1987)** | 1955 | *S. mansoni* | 47.589351 | -22.536765 |
| **WHO. Atlas of the Global Distribution of Schistosomiasis - Madagascar - Mauritius. (1987)** | 1953 | *S. mansoni* | 47.475832 | -22.189739 |
| **WHO. Atlas of the Global Distribution of Schistosomiasis - Madagascar - Mauritius. (1987)** | 1954 | *S. mansoni* | 47.828238 | -22.364933 |
| **WHO. Atlas of the Global Distribution of Schistosomiasis - Madagascar - Mauritius. (1987)** | 1955 | *S. mansoni* | 47.72136 | -22.101609 |
| **WHO. Atlas of the Global Distribution of Schistosomiasis - Madagascar - Mauritius. (1987)** | 1957 | *S. mansoni* | 47.894678 | -21.597646 |
| **WHO. Atlas of the Global Distribution of Schistosomiasis - Madagascar - Mauritius. (1987)** | 1955 | *S. mansoni* | 47.73184 | -20.988238 |
| **WHO. Atlas of the Global Distribution of Schistosomiasis - Madagascar - Mauritius. (1987)** | 1935 | *S. mansoni* | 48.181579 | -20.510791 |
| **WHO. Atlas of the Global Distribution of Schistosomiasis - Madagascar - Mauritius. (1987)** | 1959 | *S. mansoni* | 47.56558 | -20.678543 |
| **WHO. Atlas of the Global Distribution of Schistosomiasis - Madagascar - Mauritius. (1987)** | 1961 | *S. mansoni* | 48.781551 | -19.65841 |
| **WHO. Atlas of the Global Distribution of Schistosomiasis - Madagascar - Mauritius. (1987)** | 1959 | *S. mansoni* | 48.83658 | -18.537703 |
| **WHO. Atlas of the Global Distribution of Schistosomiasis - Madagascar - Mauritius. (1987)** | 1959 | *S. mansoni* | 48.470321 | -20.058293 |
| **WHO. Atlas of the Global Distribution of Schistosomiasis - Madagascar - Mauritius. (1987)** | 1956 | *S. haematobium* | 48.837047 | -16.171871 |
| **WHO. Atlas of the Global Distribution of Schistosomiasis - Madagascar - Mauritius. (1987)** | 1956 | *S. haematobium* | 47.730425 | -17.63386 |
| **WHO. Atlas of the Global Distribution of Schistosomiasis - Madagascar - Mauritius. (1987)** | 1957 | *S. haematobium* | 46.541431 | -16.439454 |
| **WHO. Atlas of the Global Distribution of Schistosomiasis - Madagascar - Mauritius. (1987)** | 1956 | *S. haematobium* | 45.856635 | -16.016655 |
| **WHO. Atlas of the Global Distribution of Schistosomiasis - Madagascar - Mauritius. (1987)** | 1951 | *S. haematobium* | 50.142867 | -15.009571 |
| **WHO. Atlas of the Global Distribution of Schistosomiasis - Madagascar - Mauritius. (1987)** | 1951 | *S. haematobium* | 47.872079 | -14.964814 |
| **WHO. Atlas of the Global Distribution of Schistosomiasis - Madagascar - Mauritius. (1987)** | 1956 | *S. haematobium* | 45.565285 | -16.580413 |
| **WHO. Atlas of the Global Distribution of Schistosomiasis - Madagascar - Mauritius. (1987)** | 1956 | *S. haematobium* | 46.120646 | -16.257579 |
| **WHO. Atlas of the Global Distribution of Schistosomiasis - Madagascar - Mauritius. (1987)** | 1959 | *S. mansoni* | 44.790163 | -23.880787 |
